# Use of Artificial Intelligence in the Classification of Upper-Limb Motion Using EEG and EMG Signals: A Review

**DOI:** 10.3390/s26051457

**Published:** 2026-02-26

**Authors:** Isabel Bandes, Yasuharu Koike

**Affiliations:** Graduate School of Engineering, Institute of Science Tokyo, Tokyo 113-8510, Japan; bandes@cns.pi.titech.ac.jp

**Keywords:** upper limbs, electroencephalogram (EEG), electromyogram (EMG), artificial intelligence, movement intent, movement classification

## Abstract

This systematic review summarizes the application of artificial intelligence (AI) in classifying upper-limb motion using Electroencephalogram (EEG) and Electromyogram (EMG) signals, focusing on the field’s progression from Traditional Machine Learning (TML) to Deep Learning (DL) architectures. Following the Preferred Reporting Items for Systematic Reviews and Meta-Analyses (PRISMA) guidelines, a search of PubMed, IEEEXplore, and Web of Science yielded 301 eligible studies published up to June 2025. The results indicate a change from classical classifiers like Linear Discriminant Analysis (LDA) and Support Vector Machines (SVMs) toward DL approaches. While Convolutional Neural Networks (CNNs) remain the most frequently implemented, emerging architectures, including Long Short-Term Memory (LSTM) networks and Transformers, have demonstrated remarkable performance. Despite the rise of DL, classical models remain highly relevant due to their robustness and efficiency. This review also identifies a heavy reliance on EEG-only modalities (60%), with only 7% of studies utilizing hybrid EEG-EMG systems, representing a potential missed opportunity for signal fusion.

## 1. Introduction

Human upper limbs are one of our primary tools for interacting with the physical world. Consequently, the congenital absence or traumatic loss of an upper limb can severely impact an individual’s capacity for daily living and independence. While prosthetic devices aim to restore lost function, their abandonment rates remain high [1]. A main factor contributing to this rejection is the steep learning curve and non-intuitive nature of conventional control mechanisms, which often fail to replicate the natural control of a native limb [2]. To address these challenges, research has increasingly moved towards Brain–Computer Interfaces (BCIs), which offer more intuitive and direct control of advanced, multi-DOF prostheses [3]. This field relies heavily on the acquisition and processing of biosignals, specifically the Electroencephalogram (EEG) and the Electromyogram (EMG) [4].

The core objective of an upper-limb motor classification BCI is to translate the user’s intent into a corresponding action in the prosthetic device by leveraging these two signals [5]. By interpreting the distinct patterns within the EEG and EMG, a BCI can command a prosthetic device to move in a way that mimics the user’s desired action [6], but it has to be able to accurately classify the signal.

EEGs measure the voltage fluctuations resulting from ionic currents within the neurons of the brain. Recorded via electrodes placed on the scalp according to standardized systems, it provides a direct view into cortical activity. Signals originating from the motor cortex are of special interest as they are modulated by motion execution and imagery [7]. The primary advantage of an EEG is its ability to capture movement intention even before a physical action is initiated [8,9]. However, it is inherently characterized by a low signal-to-noise ratio (SNR), susceptibility to artifacts (eye blinks, muscle activity, etc.), and poor spatial resolution, making the decoding of specific motor commands a significant challenge [10].

On the other hand, an EMG detects the electrical potential generated by muscle cells when they are activated. A surface EMG provides a non-invasive measure of the activity of muscle groups responsible for generating movement [11,12]. The resulting signal has a significantly higher amplitude and SNR compared to that of an EEG and is directly correlated with the force of muscle contraction. The main limitation of an EMG is that it requires a muscle to attach the sensors to, which might not always be available. Furthermore, signals can be affected by crosstalk from adjacent muscles, which can complicate the differentiation of fine motor tasks [11,13,14].

Given their complementary nature, a hybrid EEG-EMG approach combining both signals holds the potential to create a more robust and reliable upper-limb control system. The successful interpretation of these biosignals depends heavily on signal processing and classification techniques [15,16]. This is where artificial intelligence (AI) and Machine Learning (ML) have been convenient. The proliferation of these methods has enabled the development of models capable of recognizing the patterns that map raw biosignals to specific user intentions [17,18].

The classification of EEG and EEG signals for upper limbs has evolved over the years. Early research relied on statistical methods and manual feature engineering, using Traditional Machine Learning (TML) classifiers such as Linear Discriminant Analysis (LDA) and Support Vector Machines (SVMs) [19,20,21]. These models, while efficient, depended heavily on expert knowledge for feature extraction [22]. With the rise of Deep Learning (DL) and Convolutional Neural Networks (CNNs), models are now capable of performing feature learning from raw data [23,24]. Most recently, the state of the art has expanded to include temporal architectures like Long Short-Term Memory (LSTM) networks and Transformer-based models, which appear to be capable of capturing long-range dependencies in the time domain of the biosignals [25].

This review, therefore, aims to synthesize this progression, as well as analyze the application of different AI models in the classification of upper-limb movements using EEG and EMG signals. The rest of this paper is organized as follows: Section 2 outlines the Materials and Methods, explaining the search strategy and selection criteria; Section 3 presents the Results, with the quantitative data regarding model trends, dataset usage, and performance metrics; Section 4, i.e., the Discussion section, presents the possible reasons for and implications of the shift toward Deep Learning and the potential missed opportunity in signal fusion; and, finally, Section 5 offers conclusions and future research directions.

## 2. Materials and Methods

The present systematic review was conducted in accordance with the Preferred Reporting Items for Systematic Reviews and Meta-Analyses (PRISMA) guidelines [26].

### 2.1. Search Strategy

The search strategy was designed to target empirical studies that worked with artificial intelligence, biosignal processing, and upper-limb motor control. The process began with the systematic definition of the research questions and the selection of keywords, which were used for the design of the search queries to be used on the three (3) selected scientific databases: PubMed, IEEEXplore, and Web of Science. These databases were selected due to their comprehensiveness and availability.

To address the usage of AI models for the classification of EEG and EMG signals for the motion intent of upper limbs, we devised the following research questions:

- What are the predominant Machine Learning and Deep Learning trends and paradigms for classifying upper-limb motion intent using EEG and EMG signals, and what are the primary factors influencing their comparative performance?
- How do novel artificial intelligence architectures for hybrid EEG-EMG perform when used for upper-limb motion intent classification compared to more traditional Machine Learning models?

To construct the search queries, keywords were categorized into distinct conceptual groups:

- AI-Related Keywords: Artificial Intelligence, Machine Learning, Deep Learning, Neural Network, SVM, CNN, RNN, Algorithm, Methodology, and Processing, Classification.
- Biosignal-Related Keywords: EEG, Electroencephalography, EMG, and Electromyography.
- Movement-Related Keywords: Movement Prediction, Movement Intent, Motion Classification, Motor Imagery, Upper Limb, Arm, Hand, and Forearm.
- Exclusion Keywords: Review, Patients, Stroke, Diseases, Disorders, Clinical, Rehabilitation, Therapy, ECoG, Electrooculography, fMRI, Mental Disease, Pathologies, Drugs, Stimulants, and Substances.

This structured approach facilitated the formulation of search queries designed to capture the breadth of relevant research while systematically filtering out studies outside the defined scope. It was acknowledged, however, that the inclusion of some of the exclusionary terms (such as patients, clinical, etc.), while necessary to manage the high volume of research in adjacent clinical fields, carried the risk of omitting relevant foundational studies. This trade-off was deemed acceptable to maintain a focused and manageable dataset. The final search queries for each database can be found in Appendix A.

### 2.2. Database Search and Duplicate Removal

The literature search was executed across the three aforementioned databases: PubMed, IEEEXplore, and Web of Science. An initial search was conducted on 22 September 2024, performed to retrieve all relevant publications up to that date. This was subsequently updated on 10 June 2025, to ensure the inclusion of the most recent contributions to the field. After the addition of all search results, duplicates were systematically identified and removed. This initial phase yielded a total of 550 unique records.

### 2.3. Study Selection Criteria

Sets of inclusion and exclusion criteria were established to ensure that only the most relevant studies were incorporated into the final review:

#### 2.3.1. Inclusion Criteria

- Signal Modality: Studies were required to exclusively use an Electroencephalogram (EEG), an Electromyogram (EMG), or a hybrid combination of both. This criterion was established to maintain a clear focus on these signals.
- Task Focus: The primary objective of the study was the classification, detection, or recognition of signals related to upper-limb movement (actual or imagined). This ensured the direct relevance of the research to the core topic of motor decoding.
- Algorithmic Transparency: The publication’s methodology section must have described the algorithms and techniques used for signal processing and classification, enabling a detailed analysis of the AI approaches employed.
- Human Participants: Only studies conducted on human subjects were included to ensure applicability to human–machine interface design.
- Publication Timeline and Language: The search encompassed all available publication years up to June 2025 and was restricted to articles published in the English language.

#### 2.3.2. Exclusion Criteria

- Publication Type: Review papers, surveys, meta-analyses, and editorials were excluded to focus the analysis on empirical data.
- Subject Health Status: Studies involving patients or participants with neurological disorders, injuries, or any other pathological conditions were excluded. This criterion was critical for isolating the performance of AI algorithms on non-pathological signals, providing a baseline for system capabilities.
- Alternative Paradigms: Research focused on EEG/EMG paradigms not directly related to motor intent or movement recognition (e.g., sleep stage analysis, cognitive load assessment, seizure detection) was excluded.
- Alternative Biosignals: Studies employing other physiological signals such as Electrocorticography (ECoG), Electrooculography (EOG), or functional Magnetic Resonance Imaging (fMRI) for the classification of movement were excluded to maintain signal modality consistency.
- Pharmacological Influence: Studies centered on the effects of drugs, stimulants, or other substances on biosignals were deemed outside the scope of this review and were excluded.

### 2.4. Screening Process

The selection of the final papers followed a two-stage screening protocol.

#### 2.4.1. First Screening: Title and Abstract Review

The 550 unique records underwent a preliminary screening based on their titles and abstracts. During this stage, the inclusion and exclusion criteria were applied to rapidly identify and discard articles that were clearly irrelevant. This initial pass resulted in the selection of 396 articles deemed potentially relevant for full-text analysis.

#### 2.4.2. Second Screening: Full-Text Review

The full texts of the 396 selected papers were subsequently retrieved for an in-depth evaluation. The Zotero (v.6.0.37) reference manager was utilized to automate the retrieval of PDF documents. This process successfully obtained the majority of articles; however, manual search and retrieval was necessary for 66 papers. An additional 10 papers could not be accessed through our institutional resources and were consequently excluded from the review. During the full-text review, the inclusion and exclusion criteria were applied to the whole document, leading to further exclusions based on details not apparent from the title and abstract alone. Upon the completion of this second evaluation process, a final total of 301 papers were selected for inclusion in this systematic review.

**Figure 1:**
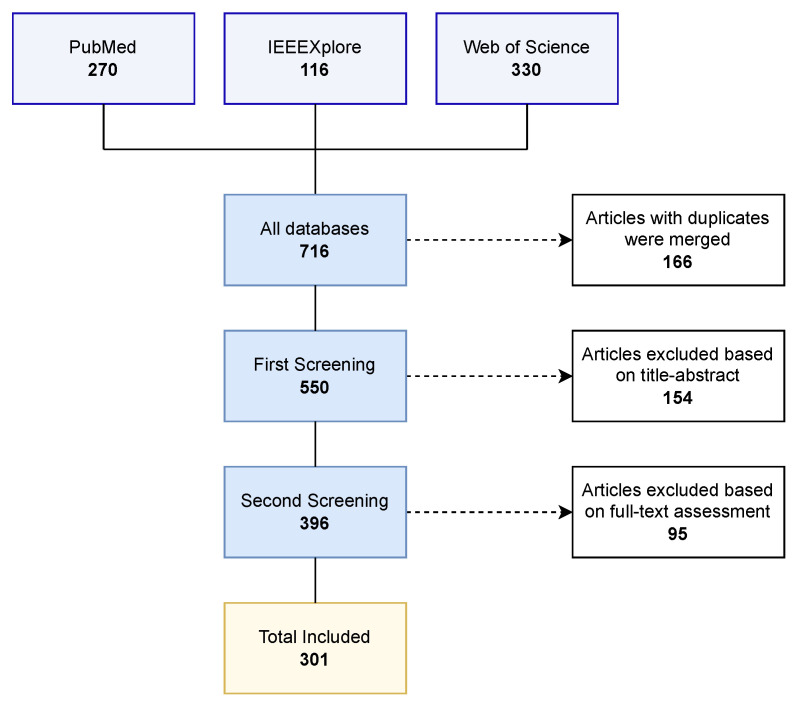
A schematic of the systematic review process, following the PRISMA guidelines, showing the number of records retrieved from each database, and the final number of included papers after the screening process.

The search, selection and screening process is shown in Figure 1.

### 2.5. Quality Assessment and Comparison Analysis

To evaluate the reporting quality of the selected studies, a custom 7-point checklist was developed, tailored to the specific requirements of reporting biosignal classification research. Each study was evaluated based on the presence or absence (1 or 0) of seven different details:

- Dataset Origin: Explicit citation of a public dataset or description of the collection protocol for private data;
- Performance Metrics: Clear reporting of the classification accuracy or relevant performance indicators;
- Number of Subjects: Explicit reporting of the participant count;
- Number of Classes: Clear specification of the number of movements classified;
- Model Architecture: A description of the classification model type;
- Feature Extraction: A description of the features used or the input characteristics for the network;
- Preprocessing: A description of the signal preprocessing steps.

The methodological quality of the included studies was evaluated based on these seven criteria, yielding an average quality score of 5.8 out of 7. This result indicates that the majority of the reviewed literature demonstrates sufficient detail to be subject to a comparative analysis.

To address the research questions, the comparative analysis focused on model architecture, where studies were categorized into TML and DL to evaluate the historical progression and performance; performance metrics, based on reported and normalized accuracy; signal modality, based on the signal (i.e., EEG, EMG, hybrid) used; and the dataset characteristics, where the data source and quantity were taken into consideration.

## 3. Results

### 3.1. General Trends

**Figure 2:**
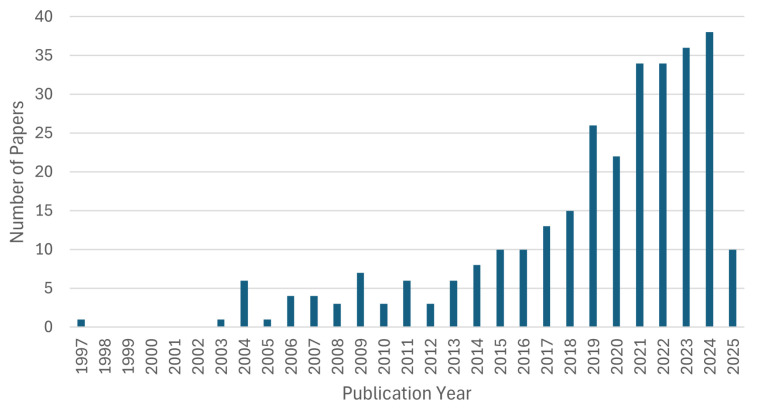
The distribution of the reviewed papers by publication year (1997–2025).

AI-based motion classification using EEGs and EMGs for upper-limb movements has expanded over the last decade. Table 1 shows some of the papers evaluated in the study. As shown in Figure 2, the publication output was small until 2014, with fewer than ten papers published per year (N= 53 from 1997 to 2014, average N= 3 papers per year). A consistent increase began around 2015, leading to a period of exponential growth starting in 2019 (N= 96 from 2015 to 2020, average N= 16 papers per year). The years from 2021 showed a faster rate of growth, accounting for over 150 of the reviewed articles (N= 152 from 2021 to 2025, N= 32 papers per year) and highlighting a surge in academic interest in this domain.

**Figure 3:**
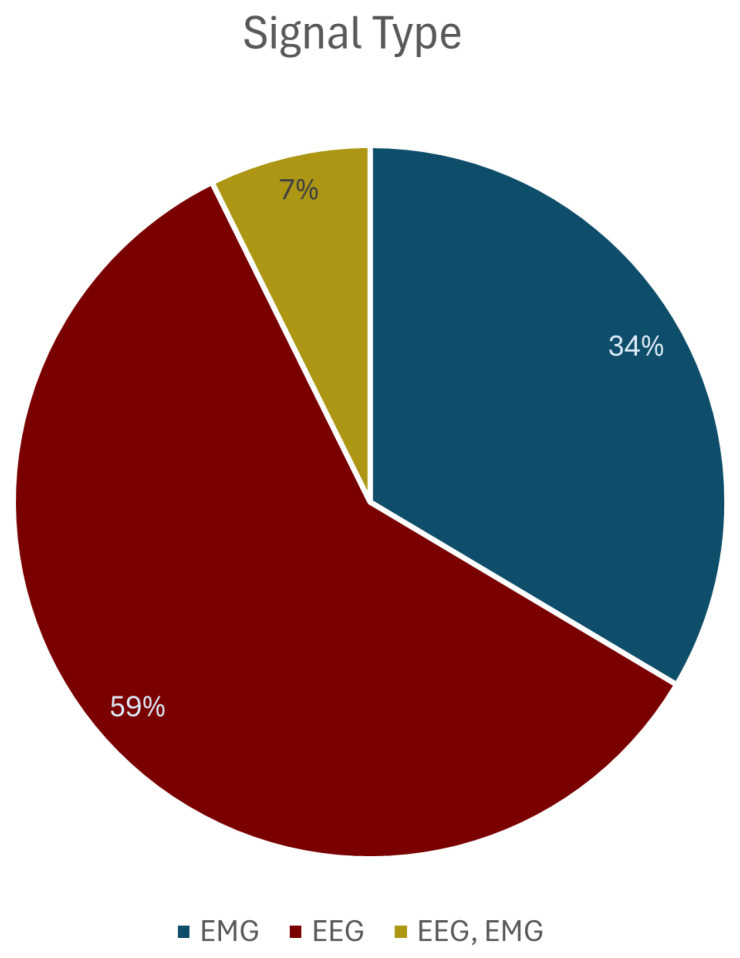
The distribution of papers based on the signal type used; EEG, EMG, or a combination of both.

The analysis of the signal modalities used reveals a strong preference for EEGs over EMGs. As detailed in Figure 3, the majority of studies (59%, N= 178) used only EEG signals. EMGs on their own were used in 34% (N= 101) of the papers, while a smaller fraction (7%, N = 22) leveraged a hybrid approach combining both EEG and EMG signals.

**Figure 4:**
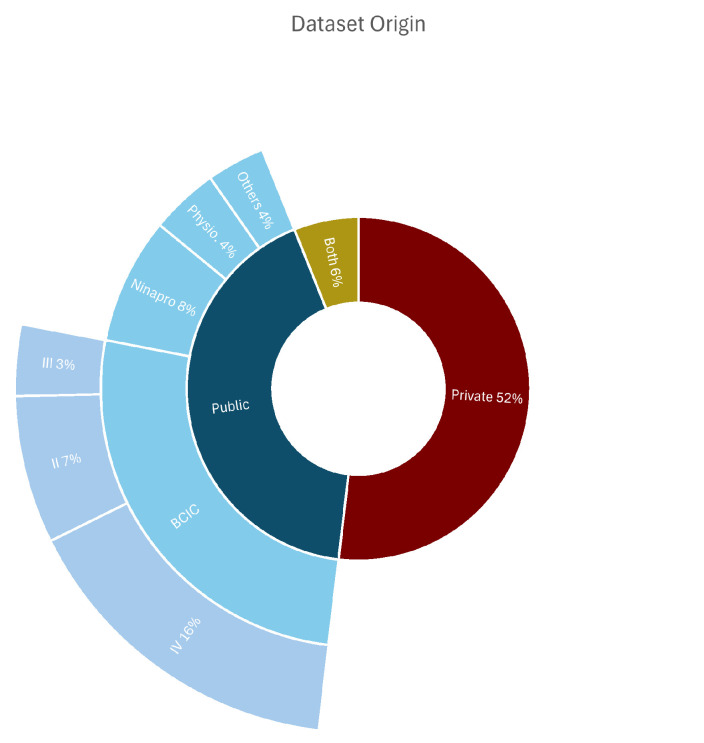
The distribution of papers based on the origin of the dataset; public, private, or a combination of both.

Regarding data sources (Figure 4), there was a relatively balanced split between private and publicly available datasets. A slight majority of studies (52%) used private datasets, while the rest relied on public ones or using a combination of both. As summarized in Table 2, among the public datasets, the BCI Competition datasets were the most frequently used, appearing in 90 articles. Other prevalent databases included Ninapro (N= 28) and Physionet (N= 13), indicating the availability and usage of common benchmarks for validating new models.

### 3.2. Methodology Trends

**Figure 5:**
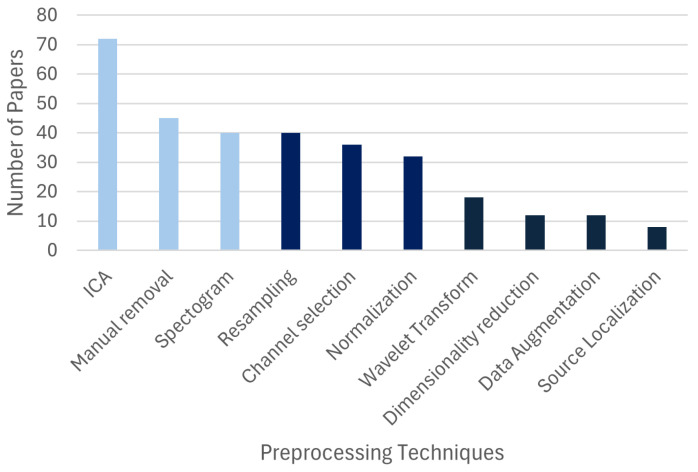
The frequencies of different signal preprocessing techniques reported across the reviewed papers.

The EEG preprocessing pipeline for the papers reviewed included different techniques used to manage artifacts and prepare the signals for analysis (Figure 5). Independent Component Analysis (ICA) (N= 72) was a popular choice for identifying physiological artifacts such as eye blinks, unrelated muscle activity, and cardiac signals [49]. Similarly, spectrograms, including Power Spectral Density (N= 40), were also used, often for enhancing the discriminability of the desired mental states. Other methods, such as the manual removal of the artifacts (N= 45) and channel selection (N= 36), were also commonly employed to optimize data quality.

**Figure 6:**
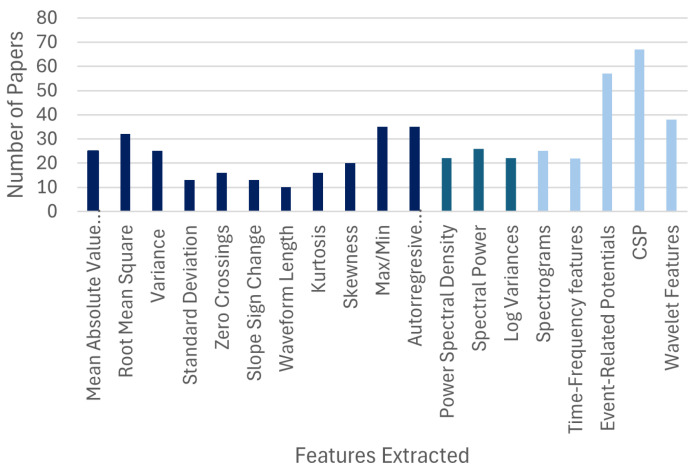
The most frequently used feature extraction techniques for EEG and EMG signals, categorized by time-domain, frequency-domain, and time-frequency methods.

In terms of feature extraction (Figure 6), several methods were employed. For EEG data specifically, spatial filters such as the Common Spatial Pattern (CSP) (N= 65) and features derived from Event-Related Potentials (ERPs) (N= 55), were highly prevalent. For both EEGs and EMGs, time-frequency features, such as Wavelet Transforms and their derivations (N= 38), were also a popular choice. Among traditional frequency-domain features, Power Spectral Density (PSD) and other measures of spectral power were frequently used (N= 36). For time-domain features, Root Mean Square (RMS) (N= 32), Mean Absolute Value (MAV) (N= 25), and Variance (N= 25) were the most common. It is notable that many papers using Deep Learning models did not report a distinct feature extraction step, as these architectures often learn relevant features directly from the data.

**Figure 7:**
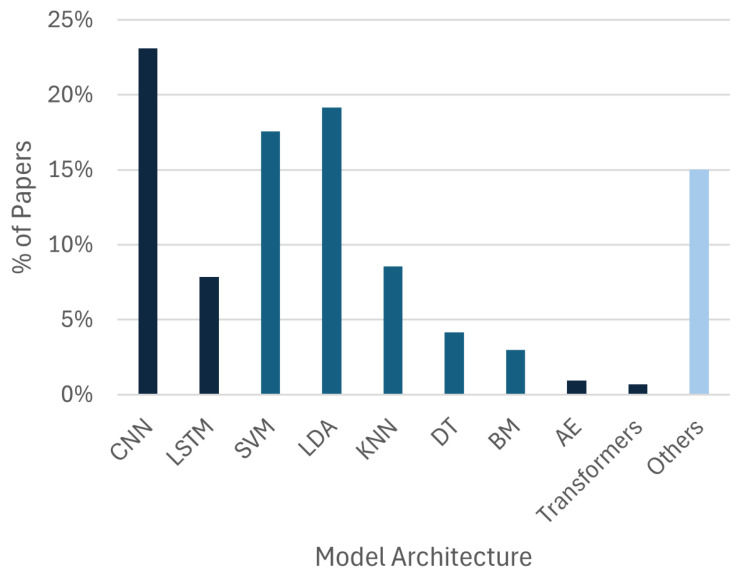
The distribution of AI model architectures across the reviewed studies. Quantities sum to over 301, as many studies implemented multiple models for comparison or improvement purposes.

Figure 7 details the AI model distribution. Convolutional Neural Networks (CNNs) are the most implemented architecture (N=107). However, Linear Discriminant Analysis (LDA, N=83) and Support Vector Machines (SVMs, N=76) still have a strong presence, often used in the same studies as benchmark comparison. Long Short-Term Memory (LSTM) networks (N=34) also appear frequently, used for their ability to model temporal dependencies. More recently, architectures such as Autoencoders and Transformers have gained traction. However, the number of studies focusing on those types of models were small for the present review (N=4 and N=3, respectively).

**Figure 8:**
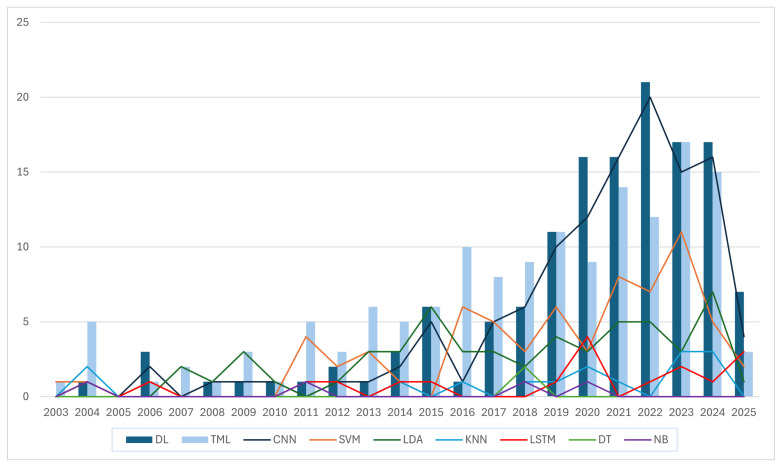
The number of papers according to the AI model used per year, demonstrating a progressive shift from traditional models like LDA and SVM towards CNNs since around 2016.

The temporal evolution of model usage, depicted in Figure 8, reveals a progressive shift. In the years prior to 2016, classical models like LDA and SVM were the standard. A change began around 2017, where the application of Deep Learning started to spread. The authors note that, while the selected search keywords and the 1997 to early 2025 window may have excluded state-of-the-art papers, the data shows a trend toward DL-based models.

### 3.3. Performance Analysis

**Figure 9:**
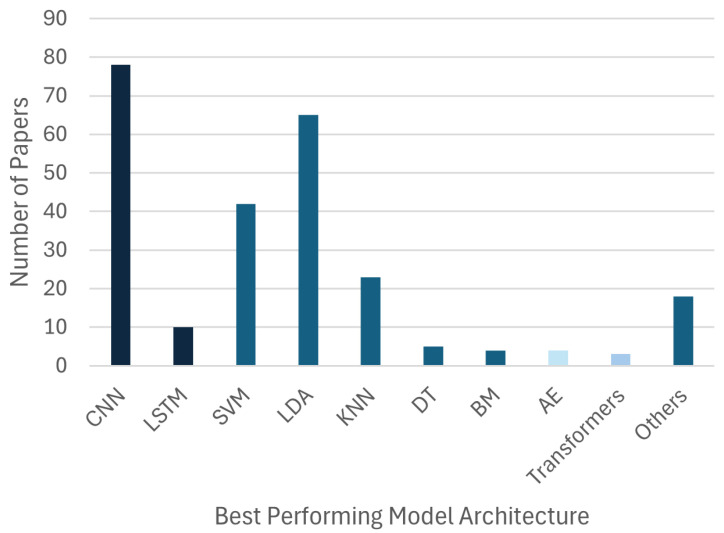
The number of papers in which the AI model used was reported as the “best performing” in the corresponding studies.

When authors explicitly identified a single “best performing model” in their comparative analyses, the results reinforce the dominance of Deep Learning (Figure 9). CNNs were most frequently reported as the top-performing model (N= 85), followed by SVMs (N= 42) and LDA (N= 65). This suggests that while traditional models are highly effective, CNNs increasingly achieve superior performance in direct comparisons. While their representation in the current review is sparse, likely due to the search strategies used, some results on Transformers and Autoencoders are notable. Hassanpour et al. achieved 90.21% EEG classification accuracy using Stacked Sparse Autoencoders [50], and Basturk et al. applied Deep Autoencoder Networks for movement prediction [51]. Among Transformer-based approaches, Li et al. reported 94.96% accuracy for EEG classification [52], while Ng et al. achieved 85.38% using an attention-based model [53], and Mao et al. proposed transformer-based models to address cross-subject variability [54].

**Figure 10:**
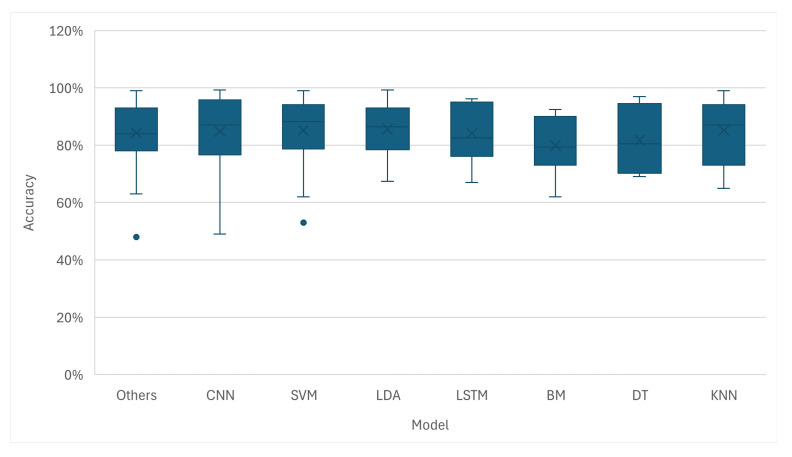
The range and median of reported classification accuracies for different AI models, comparing the performance spread of different architectures.

The box plot in Figure 10 illustrates the distribution of reported accuracies for different model architectures. Deep Learning models like CNNs and LSTMs demonstrate high median accuracies, with many studies achieving results exceeding 90%. At the same time, classical models like LDA and SVM also show very strong performances, with median accuracies well above 80% and many reported results exceeding 95%. This might indicate that while Deep Learning defines the state of the art, traditional methods remain powerful and highly competitive.

**Figure 11:**
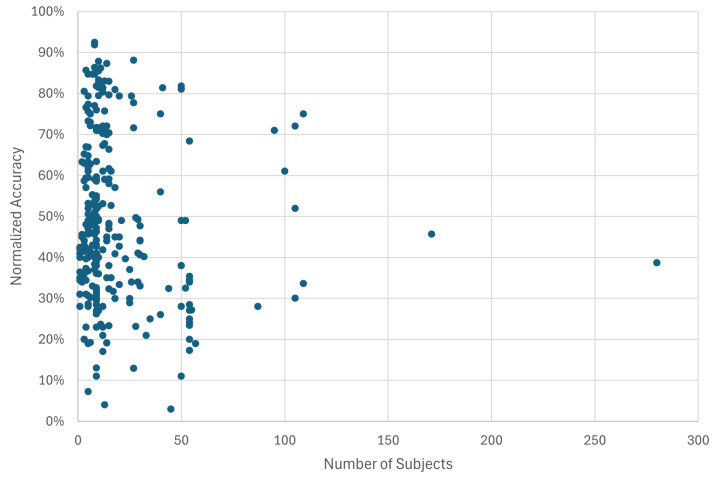
Normalized accuracy according to dataset size.

**Figure 12:**
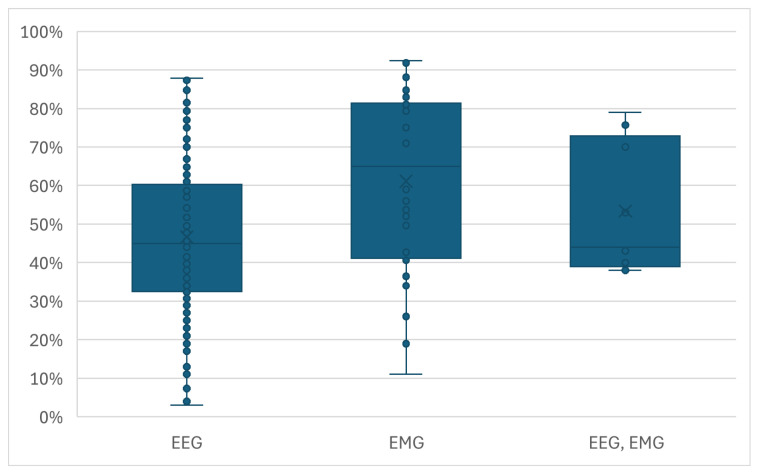
The distribution of normalized accuracies across the different signal types.

**Figure 13:**
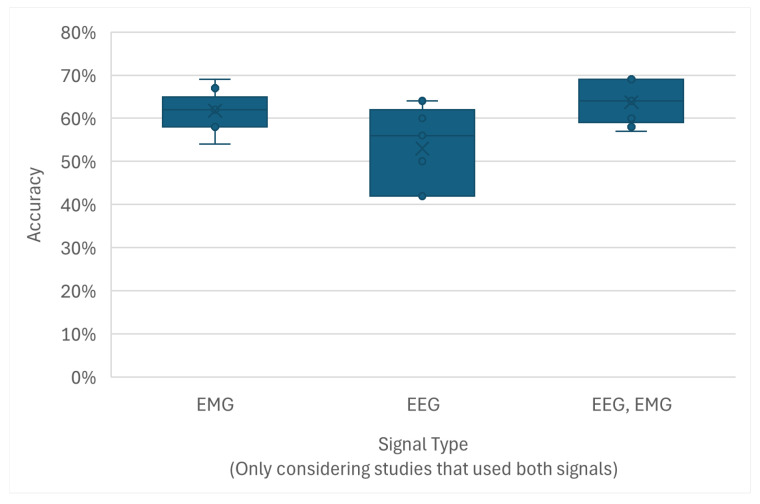
Performance gain analysis within hybrid studies. The figure displays the distribution of normalized accuracies for the individual signal components (EEG-only and EMG-only) compared to the fused hybrid signal reported in the same studies. Not all hybrid studies reported baseline accuracies for single modalities.

**Figure 14:**
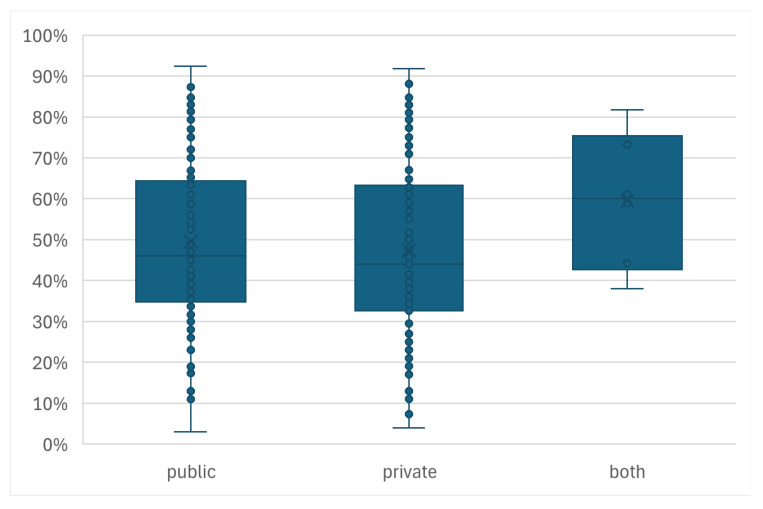
The distribution of the normalized accuracies based on whether the study used a private dataset, a public dataset, or a combination of both datasets.

Further analysis revealed that classification accuracy is not strongly correlated with a single experimental variable. To ensure a fair comparison across studies with varying complexity, ranging from binary classification to tasks with over 50 movements, the quantitative analysis presented in Figure 11, Figure 12, Figure 13 and Figure 14 utilizes normalized accuracy instead of reported overall accuracy. This metric is defined as the reported accuracy minus the theoretical random chance level (1/N, where *N* is the number of classes). This adjustment allows for an evaluation of the model’s predictive power beyond random guessing. It should be noted that this calculation assumes a balanced class distribution (1/N), which is a limitation of this review, as not all included studies explicitly reported class balance ratios.

Dataset Size (Figure 11): No direct correlation was observed between the number of subjects in a study and the reported accuracy. High accuracies were reported for studies with both small (N < 10) and large (N > 100) participant pools. This suggests that factors like model choice, data quality, and experimental design are more dominant drivers of performance than dataset size alone.

Signal Type (Figure 12 and Figure 13): High normalized classification accuracies were achieved across all signal modalities. The median accuracies for EMG, EEG, and hybrid EEG-EMG systems were all high, though with wide Variance. Studies using EMGs tended to report slightly higher median accuracies than those using EEGs, and the hybrid approach showed an improvement over using EEGs alone, suggesting a potential benefit in signal fusion.

Recent studies exploring hybrid EEG-EMG paradigms demonstrate that signal fusion often yields superior performance compared to single-modality approaches, particularly when leveraging Deep Learning architectures [55]. For instance, Aly and Youssef achieved 95.20% accuracy in classifying hand and wrist motions by employing a hybrid CNN-LSTM model that fuses features without the need for manual engineering [29]. Similarly, Tayeb et al. reported high offline decoding accuracies (up to 93.75%) using CNNs within their ‘Gumpy’ hybrid BCI toolbox [56]. These results reinforce the observation that combining the pre-movement intent captured by EEGs with the muscular activation data from EMGs creates a more robust control signal, a conclusion further supported by Chowdhury et al. [31].

Recent methodologies have also adopted learning strategies where one modality enhances the decoding of the other [28,57]. Das et al. proposed a hierarchical approach that first estimates EMG signals from EEGs using linear regression before predicting finger movements [30]. Cho et al. introduced a dual-stage framework where EMG-based muscle synergy labels guide the training of a CNN to extract relevant EEG features, improving motor imagery classification [58]. Other approaches include the work of Xi et al., who used EMG bursts to guide the sampling of EEG signals for enhanced coherence analysis [27,59]. These advanced fusion techniques highlight a potential advantage in using the complementary nature of EEGs and EMGs for signal enhancement.

Dataset Origin (Figure 14): The source of the data was also not a clear predictor of final accuracy. The distributions of normalized accuracies for studies using public and private datasets were very similar, indicating that decent performance is achievable with either resource.

## 4. Discussion

The review of the papers revealed a shift in the classification of upper-limb motion. While the field was historically dominated by classical classifiers like LDA and SVM, there has been an exponential growth in research output since 2021, driven largely by the adoption of Deep Learning architectures.

### 4.1. Trend Shift

The most notable trend identified in this review is the decisive pivot from Traditional Machine Learning (TML) towards Deep Learning (DL) architectures, including various architectures like CNNs and LSTMs, which have increasingly being implemented and, frequently, have ended up as the best-performing models in recent years.

This shift is largely attributable to the fundamental difference in how these models process information. The surge in DL usage is driven by the capacity of these architectures to perform automatic feature extraction [60]. Unlike classical TML models, which depend heavily on handcrafted features, Neural Networks can learn representations directly from raw or minimally processed biosignals [61]. This capability reduces reliance on manual feature engineering and allows the models to uncover complex and non-linear patterns that traditional methods might miss, often leading to superior classification accuracies, as illustrated in Figure 9 and Figure 10.

However, the continued prevalence and strong performance of classical models is still relevant [62]. Their sustained relevance stems from their robustness, computational efficiency, and interpretability. For many applications, especially those with limited training data or those requiring real-time processing on low-power hardware, TML provides a reliable, lightweight option [63]. Ultimately, the results suggest that the field might be developing a more diverse toolkit, where the choice between Traditional Machine Learning and Deep Learning is tailored to the specific constraints of the task [64] rather than just performance.

### 4.2. Signal Preference

The preference for EEGs over EMGs or hybrid systems reflects the field’s focus on decoding motor intent directly from the brain. The primary advantage of EEG is its ability to capture signals related to motor imagery, making it viable for users who lack the residual muscle activity required for EMGs [65]. However, this focus comes at the cost of addressing the inherent drawbacks of EEGs, specifically their low signal-to-noise ratios and high susceptibility to artifacts.

Concurrently, the limited utilization of hybrid EEG-EMG systems (7%) represents a significant missed opportunity [66]. Although the results presented in Figure 12 and Figure 13 indicate only a marginal accuracy improvement for the few hybrid studies compared to those using EEGs alone, the theoretical potential is substantial [28]. Combining the pre-movement intentionality captured by EEGs with the clear, high-SNR signal of executed muscle contraction from EMGs could yield BCI systems that are more robust, intuitive, and less prone to error [61]. The barely growing numbers of studies in this specific area highlights a notable gap in the field [67]. The missed opportunity in signal combination lies not just in combining signals but in the application of Deep Learning-based fusion architectures. Advanced multimodal networks capable of learning joint representations and potentially mapping the neural intent to the resulting muscle activation hold the potential to achieve the desired robustness for hybrid BCIs.

### 4.3. Dataset Size

Another key finding of this review is the lack of a clear correlation between the reported classification accuracy and the number of subjects (Figure 11) or dataset origin (Figure 14). While it might be hypothesized that studies utilizing public datasets or larger participant pools would yield more reliable results [33], the data does not reflect this trend. This discrepancy suggests several underlying issues.

Primarily, the field suffers from a lack of standardization in evaluation protocols. Diversity in experimental paradigms, signal processing, cross-validation techniques, data segmentation methods, and performance metrics makes direct comparison of reported accuracies challenging and potentially misleading [68]. Furthermore, while larger datasets generally improve model generalization [69], the high accuracies reported for smaller datasets may reflect overfitting on subject-specific models that are less influenced by subject inter-variability [11]. Consequently, these results do not guarantee that such models will generalize effectively to new users [70]. The lack of correlation between dataset size and performance likely points to confounding variables such as task complexity and model capacity. For instance, a study reporting 95% accuracy on a binary classification task (open/close hand, move/rest, etc) is fundamentally different from a study achieving 80% on a 50-class finger movement task, and grouping these studies solely by participant count obscures this distinction [71]. Furthermore, the high accuracies reported in some studies with small participant pools raise concerns regarding model capacity and overfitting. The models may be effectively “memorizing” the small dataset rather than learning generalized motor features, resulting in inflated performance metrics that would likely degrade in cross-subject validation [72]. Finally, it must be noted that the use of normalized accuracy in this review assumes a balanced class distribution. In practical BCI scenarios, however, class imbalance is common. The rest state often has higher representations compared to active tasks, for example, which affects metric interpretation. For instance, for a dataset where the resting state constitutes 90% of the samples, a trivial model that predicts “Rest” for every input achieves a misleadingly high accuracy of 90%, despite having no discerning cognition on the user’s actual motor intent [73,74]. This represents a limitation in accuracy report and comparison; without the inclusion of metrics such as the F1-score, Precision–Recall curves, or Cohen’s Kappa (κ), comparisons of model performance remain superficial and potentially biased toward models that favor the majority class.

From a statistical perspective, however, the need for larger datasets, balanced or imbalanced, remains critical to ensure the reliability and validity of results. Findings derived from small sample sizes often lack statistical power, leading to wider confidence intervals around reported metrics. Although a performance metric may appear high, it may be less certain and unrepresentative of the broader population [75]. Larger datasets are crucial not only for increasing statistical confidence but also for capturing subject variability, ensuring that models are robust and unbiased across different individuals.

### 4.4. Study Limitations

Despite the comprehensive nature of this systematic review, several limitations must be acknowledged. First, the search strategy, while designed to be thorough, may have inadvertently excluded relevant studies. The use of exclusionary keywords, particularly those related to clinical conditions and patient populations, was necessary to manage the high volume of adjacent research and to establish a baseline of algorithmic performance without the confounding influence of pathological signal degradation. However, this approach may have omitted relevant studies that utilized non-pathological data from clinical populations or studies where keywords differed in terminology.

Additionally, while multiple other biosignal modalities are available for motor control, the scope of the present review was limited to EEGs and EMGs. While other modalities such as functional Magnetic Resonance Imaging (fMRI), functional Near-Infrared Spectroscopy (fNIRS), and Electrooculography (EOG) offer valuable insights into motor control, they were excluded mainly for practical reasons [76]. fMRI, despite its high spatial resolution, requires bulky, non-portable equipment that lowers its feasiblility for real-time, wearable applications. Similarly, while fNIRS is more portable, it measures hemodynamic responses, which typically exhibit a temporal lag of several seconds compared to electrical signals, which contrasts with the real-time responsiveness desired in upper-limb prosthetics [77,78]. Furthermore, signals like EOG are frequently treated as artifacts to be removed in motor decoding pipelines rather than primary control signals for limb mechanics [79].

The analysis performed is also constrained by the quality and reporting standards of the included papers. As highlighted previously, the lack of standardization in evaluation protocols and performance metrics complicates direct quantitative comparisons. The observation that high reported accuracies often do not correlate with factors like sample size is a direct consequence of this heterogeneity. Readers should interpret reported performance figures within the context of these methodological inconsistencies [80]. Additionally, the use in the present review of normalized accuracy (reported accuracy minus theoretical chance level) as a performance metric carries limitations on its own. The subtraction-based metric was adopted as a necessary method to account for the variation in chance levels; however, it should be interpreted with caution, as this metric mathematically favors studies with higher numbers of classes and does not account for class imbalance.

The review also focuses exclusively on studies published in the English language, which may have excluded contributions from researchers publishing in other languages. Additionally, while a custom quality checklist was assessed to ensure methodological reproducibility (5.8/7), a formal Risk of Bias assessment was not performed; given that the majority of reviewed studies are engineering-centered rather than clinical trials, a quality assessment was deemed sufficient.

Finally, while this review provides a robust synthesis of the existing literature, it is inherently time-sensitive. The field of AI is evolving rapidly, with new architectures and methodologies emerging frequently. Therefore, the trends and conclusions drawn here represent the state of the field up to the final search date for this review.

### 4.5. Future Directions

To advance the field, several key areas should be prioritized. First, there is a pressing need for the community to adopt standardized reporting guidelines and evaluation metrics to enable meaningful comparisons between studies and provide a clearer picture of progress. Research should also prioritize the development of models capable of generalizing across subjects, moving away from subject-specific training toward techniques such as transfer learning and domain adaptation to create more robust, user-independent systems. The potential of fusing EEG and EMG signals remains underexplored [81]; future work should focus on developing novel AI architectures specifically designed to leverage complementary information from both modalities [2,82].

Beyond specific architectures, the field stands to benefit significantly from the integration of emerging “Modern AI” paradigms. While the currently reviewed literature is dominated by CNNs and LSTMs, the application of Large Foundation Models (LFMs) and Generative Pre-trained Transformers (GPTs) represents a future opportunity. These models, though originally designed for natural language, possess valid capabilities for time-series analysis and code generation, potentially aiding in the automated design of BCI pipelines or the interpretation of complex user intents.

Furthermore, true robustness will likely require a shift toward Multimodal AI that extends beyond simple signal fusion. Future systems should look to integrate biosignals (EEGs/EMGs) with computer vision (e.g., identifying the object that the user intends to grasp) and inertial data to create context-aware prosthetic controllers. Finally, as Deep Learning models grow in complexity, the “black box” problem becomes a barrier to clinical adoption. The implementation of Explainable AI (XAI) techniques is essential to decode what the model is learning, ensuring that classification is based on neurophysiological features rather than artifacts.

## 5. Conclusions

The findings of this systematic review showcase a shift in the application of artificial intelligence for classifying motion intent from EEG and EMG signals. While the impulse to test the latest architectures is a natural characteristic of research, the field’s transition from traditional models toward Deep Learning, particularly CNNs, is substantiated by their capacity to automatically learn complex features from raw biosignals. However, it is worth noting that novelty is not always synonymous with superiority; classical models remain robust benchmarks, and the choice of model should be driven by application constraints rather than the allure of state-of-the-art options.

Despite the impressive progress in classification accuracy, critical challenges persist. The lack of standardized protocols and evaluation metrics across studies complicates direct comparisons and can obscure the true rate of advancement. Furthermore, the prevailing focus on single-signal decoding overlooks the potential of hybrid EEG-EMG systems.

To move from laboratory success towards real-world application, future research should prioritize standardized reporting to foster meaningful comparisons and develop models that generalize across subjects to minimize calibration. Ultimately, the goal should not be only to achieve the highest possible accuracy on a dataset but to create interfaces that are able to restore function and independence to the user, as well as open up possibilities for fully intuitive prosthetic control.

## Figures and Tables

**Table 1:** Biosignal studies categorized by sgnal type, randomly selected from the top 20% in terms of performance.

Authors	Year	Signal	Data Type	Dataset	Subj.	Classes	Model	Acc.
Signal: Both (EEG + EMG)								
Xia et al. [27]	2020	Both	Private	Private	5	3	SVM	83.5%
Liang et al. [28]	2020	Both	Private	Private	4	5	LDA	91.7%
Aly et al. [29]	2023	Both	Private	Private	4	5	LSTM	95.2%
Das et al. [30]	2023	Both	Private	Private	6	5	LSTM	84.2%
Chowdhury et al. [31]	2019	Both	Private	Private	16	2	SVM	92.8%
Signal: EEG								
Lopez-Larraz et al. [32]	2014	EEG	Private	Private	6	7	LDA	74%
Zhang et al. [33]	2019	EEG	Public	Physionet	103	2	LSTM	98.3%
Degrimenci et al. [34]	2024	EEG	Public	Kaya	8	6	BM	75%
Suwannarat et al. [35]	2018	EEG	Private	Private	11	3	LDA	96%
Liu et al. [36]	2023	EEG	Private	Private	10	4	SVM	83.3%
Signal: EMG								
Abbas et al. [37]	2024	EMG	Private	Private	35	8	SVM	97.1%
Pang et al. [38]	2015	EMG	Public	Ninapro	10	41	LDA	83%
Pizzolato et al. [39]	2017	EMG	Public	Ninapro	10	41	RF	74%
Chen et al. [40]	2013	EMG	Private	Private	6	10	SVM	97.9%
Gupta et al. [41]	2019	EMG	Private	Private	12	6	NN	98%

**Table 2:** The most used public datasets.

Dataset	No. Subjects	No. Movements	Modality	No. Channels	N of Papers
Ninapro (DB1) [42]	27	52	EMG	12	28
Ninapro (DB2) [42]	40	49	EMG	12	
BCI Competition IV [43]	9	4	EEG	22	55
BCI Competition III [44]	5	2	EEG	118	11
BCI Competition II [45]	1	2	EEG	3	24
Physionet [46]	109	3	EEG	64	13
GigaDB [47]	25	11	EEG/EMG	60/7	8
OpenBMI [48]	54	2	EEG	62	6

## Data Availability

The original contributions presented in this study are included in the article. Further inquiries can be directed to the corresponding author.

## Abbreviations

The following abbreviations are used in this manuscript:

AE	Autoencoder
AI	Artificial Intelligence
BCI	Brain–Computer Interface
BCIC	Brain–Computer Interface Competition
BM	Bayesian Methods
CNN	Convolutional Neural Network
CSP	Common Spatial Pattern
DL	Deep Learning
DOF	Degrees of Freedom
DT	Decision Tree
ECOG	Electrocorticography
EEG	Electroencephalogram
EMG	Electromyogram
EOG	Electrooculography
ERP	Event-Related Potential
fMRI	functional Magnetic Resonance Imaging
fNIRS	functional Near-Infrared Spectroscopy
GPT	Generative Pre-trained Transformer
ICA	Independent Component Analysis
KNN	K-Nearest Neighbors
LDA	Linear Discriminant Analysis
LFM	Large Foundation Models
LSTM	Long Short-Term Memory
MAV	Mean Absolute Value
ML	Machine Learning
NB	Naive Bayes
NN	Neural Network
PRISMA	Preferred Reporting Items for Systematic Reviews and Meta-Analyses
PSD	Power Spectral Density
RF	Random Forest
RMS	Root Mean Square
RNN	Recurrent Neural Network
SNR	Signal-to-Noise Ratio
SVM	Support Vector Machine
TML	Traditional Machine Learning
XAI	Explainable Artificial Intelligence

## Appendix A

The search queries used for each database are detailed below: Web of Science: (((EEG OR Electroencephalography OR EMG OR Electromyography) AND (“Movement Classification” OR “Movement Intent” OR “Movement Prediction” OR “Motor Imagery”) AND (“Upper Limb” OR “Upper-limb” OR Arm OR Hand OR Forearm) AND (Algorithm OR Methodology OR Processing OR Classification OR “Neural Network*” OR SVM OR CNN OR RNN OR “Deep Learning” OR “Machine Learning”)) NOT (Review OR Patient* OR Stroke OR Disease* OR Disorder* OR Clinical OR Rehabilitation OR Therapy OR ECoG OR Electrooculography OR fMRI OR “Mental Disease” OR Patholog* OR Drug* OR Stimulant* OR Substance*)) IEEEXplore: (ALL:(EEG OR Electroencephalography OR EMG OR Electromyography) AND ALL:(“Movement Classification” OR “Movement Intent” OR “Movement Prediction” OR “Motor Imagery”) AND ALL:(“Upper Limb” OR “Upper-limb” OR Arm OR Hand OR Forearm) AND ALL:(Algorithm OR Methodology OR Processing OR Classification OR “Neural Network*” OR SVM OR CNN OR RNN OR “Deep Learning” OR “Machine Learning”)) NOT (ALL:(Review OR Patient* OR Stroke OR Disease* OR Disorder* OR Clinical OR Rehabilitation OR Therapy OR ECoG OR Electrooculography OR fMRI OR “Mental Disease” OR Patholog* OR Drug* OR Stimulant* OR Substance*)) PubMed: ((EEG[tiab] OR Electroencephalography[tiab] OR EMG[tiab] OR Electromyography[tiab]) AND (“Movement Classification”[tiab] OR “Movement Intent”[tiab] OR “Movement Prediction”[tiab] OR “Motor Imagery”[tiab]) AND (“Upper Limb”[tiab] OR “Upper-limb”[tiab] OR Arm[tiab] OR Hand[tiab] OR Forearm[tiab]) AND (Algorithm[tiab] OR Methodology[tiab] OR Processing[tiab] OR Classification[tiab] OR “Neural Network*”[tiab] OR SVM[tiab] OR CNN[tiab] OR RNN[tiab] OR “Deep Learning”[tiab] OR “Machine Learning”[tiab])) NOT ((Review[Publication Type] OR Patient*[tiab] OR Stroke[tiab] OR Disease*[tiab] OR Disorder*[tiab] OR Clinical[tiab] OR Rehabilitation[tiab] OR Therapy[tiab] OR ECoG[tiab] OR Electrooculography[tiab] OR fMRI[tiab] OR “Mental Disease”[tiab] OR Patholog*[tiab] OR Drug*[tiab] OR Stimulant*[tiab] OR Substance*[tiab]))
